# Thoracic and cardiovascular surgeries in Japan during 2019: Annual report by the Japanese Association for Thoracic Surgery

**DOI:** 10.1007/s11748-023-01945-4

**Published:** 2023-07-20

**Authors:** Committee for Scientific Affairs, The Japanese Association for Thoracic Surgery, Kenji Minatoya, Yukio Sato, Yasushi Toh, Tomonobu Abe, Shunsuke Endo, Yasutaka Hirata, Michiko Ishida, Hisashi Iwata, Takashi Kamei, Nobuyoshi Kawaharada, Shunsuke Kawamoto, Kohji Kohno, Hiraku Kumamaru, Goro Matsumiya, Noboru Motomura, Rie Nakahara, Morihito Okada, Hisashi Saji, Aya Saito, Hideyuki Shimizu, Kenji Suzuki, Hirofumi Takemura, Tsuyoshi Taketani, Hiroya Takeuchi, Wataru Tatsuishi, Hiroyuki Yamamoto, Takushi Yasuda, Masayuki Watanabe, Naoki Yoshimura, Masanori Tsuchida, Yoshiki Sawa

**Affiliations:** 1 Committee for Scientific Affairs, The Japanese Association for Thoracic Surgery, Tokyo, Japan; 2 Department of Cardiovascular Surgery, Graduate School of Medicine, Kyoto University, Kyoto, Japan; 3 Department of Thoracic Surgery, University of Tsukuba, Tsukuba, Japan; 4 Department of Gastroenterological Surgery, National Hospital Organization, Kyushu Cancer Center, Fukuoka, Japan; 5 Division of Cardiovascular Surgery, Department of General Surgical Science, Gunma University, Maebashi, Japan; 6 Thoracic Surgery, Jichi Medical University Saitama Medical Center, Omiya, Japan; 7 Department of Cardiac Surgery, The University of Tokyo Hospital, Tokyo, Japan; 8 Cardiac Surgery, Handa City Hospital, Aichi, Japan; 9 Department of General Thoracic Surgery, Gifu University Hospital, Gifu, Japan; 10 Department of Surgery, Graduate School of Medicine, Tohoku University, Sendai, Japan; 11 Department of Cardiovascular Surgery, Sapporo Medical University School of Medicine, Sapporo, Japan; 12 Department of Cardiovascular Surgery, Tohoku Medical and Pharmaceutical University Hospital, Sendai, Japan; 13 Department of Gastrointestinal Tract Surgery, Fukushima Medical University, Fukushima, Japan; 14 Department of Healthcare Quality Assessment, Graduate School of Medicine, The University of Tokyo, Tokyo, Kagoshima Japan; 15 Department of Cardiovascular Surgery, Chiba University Graduate School of Medicine, Chiba, Japan; 16 Department of Cardiovascular Surgery, Toho University Sakura Medical Center, Chiba, Japan; 17 Division of Thoracic Surgery, Tochigi Cancer Center, Toshigi, Japan; 18 Surgical Oncology, Hiroshima University, Hiroshima, Japan; 19 Department of Chest Surgery, St. Marianna University School of Medicine, Kawasaki, Japan; 20 Department of Surgery, Graduate School of Medicine, Yokohama City University, Yokohama, Japan; 21 Department of Cardiovascular Surgery, Keio University, Tokyo, Japan; 22 Department of General Thoracic Surgery, Juntendo University School of Medicine, Tokyo, Japan; 23 Department of Cardiovascular Surgery, Kanazawa University, Kanazawa, Japan; 24 Department of Cardiovascular Surgery, Mitsui Memorial Hospital, Tokyo, Japan; 25 Department of Surgery, Hamamatsu University School of Medicine, Shizuoka, Japan; 26 Division of Cardiovascular Surgery, Department of General Surgical Science, Gunma University, Maebashi, Japan; 27 Department of Healthcare Quality Assessment, Graduate School of Medicine, The University of Tokyo, Tokyo, Japan; 28 Department of Surgery, Kindai University Faculty of Medicine, Osaka, Japan; 29 Department of Gastroenterological Surgery, Cancer Institute Hospital, Tokyo, Japan; 30 Department of Thoracic and Cardiovascular Surgery, Graduate School of Medicine, University of Toyama, Toyama, Japan; 31 Division of Thoracic and Cardiovascular Surgery, Niigata University Graduate School of Medical and Dental Sciences, Niigata, Japan; 32 Osaka University Graduate School of Medicine/Osaka Police Hospital, Osaka, Japan

Since 1986, the Japanese Association for Thoracic Surgery has conducted annual thoracic surgery surveys throughout Japan to determine statistics on the number of procedures performed by surgical categories. Herein, we summarize the results of the association’s annual thoracic surgery surveys in 2019. We regret that, for various reasons, this report has been delayed to 2023.

Adhering to the norm thus far, thoracic surgery had been classified into three categories, including cardiovascular, general thoracic, and esophageal surgeries, with patient data for each group being examined and analyzed. We honor and value all members’ continued professional support and contributions.

Incidence of hospital mortality was included in the survey to determine nationwide status, which has contributed to Japanese surgeons’ understanding of the present status of thoracic surgery in Japan while helping in surgical outcome improvements by enabling comparisons between their work and that of others. This approach has enabled the association to gain a better understanding of present problems and prospects, which is reflected in its activities and member education.

The 30-day mortality (also known as *operative mortality*) is defined as death within 30 days of surgery, regardless of the patient’s geographic location, including post-discharge from the hospital. *Hospital mortality* is defined as death within any time interval following surgery among patients yet to be discharged from the hospital.

Transfer to a nursing home or a rehabilitation unit is considered hospital discharge unless the patient subsequently dies of complications from surgery, while hospital-to-hospital transfer during esophageal surgery is not considered a form of discharge. In contrast, hospital-to-hospital transfer 30 days following cardiovascular and general thoracic surgeries are considered discharge given that National Clinical Database (NCD)-related data were used in these categories.

## Survey abstract

All data on cardiovascular, general thoracic, and esophageal surgeries were obtained from the NCD. In 2018, the data collection method for general thoracic and esophageal surgeries had been modified from self-reports using questionnaire sheets following each institution belonging to the Japanese Association for Thoracic Surgery to an automatic package downloaded from the NCD in Japan.

The data collection related to cardiovascular surgery (initially self-reported using questionnaire sheets in each participating institution up to 2014) changed to downloading an automatic package from the Japanese Cardiovascular Surgery Database (JCVSD), which is a cardiovascular subsection of the NCD in 2015.

## Final report: 2019

### (A) Cardiovascular surgery

We are extremely pleased with the cooperation of our colleagues (members) in completing the cardiovascular surgery survey, which has undoubtedly improved the quality of this annual report. We are truly grateful for the significant efforts made by all participants within each participating institution in completing the JCVSD/NCD.

**Fig. 1:**
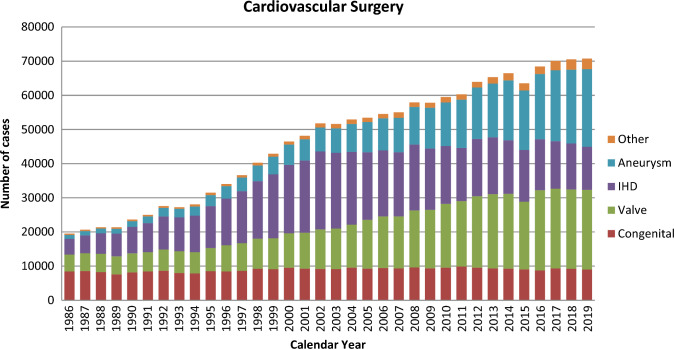
Cardiovascular surgery. *IHD* ischemic heart disease

Figure 1 illustrates the development of cardiovascular surgery in Japan over the past 33 years. Aneurysm surgery includes only surgeries for thoracic and thoracoabdominal aortic aneurysms. Extra-anatomic bypass surgery for thoracic aneurysm and pacemaker implantation have been excluded from the survey since 2015. Assist device implantations were not included in the total number of surgical procedures but were included in the survey.

A total of 70,769 cardiovascular surgeries, including 84 heart transplants, had been performed in 2019, with a 0.32% increase compared to that in 2018 (*n* = 70,537).

Compared to data for 2018 [1] and 2009 [2], data for 2019 showed 2.7% (9006 vs 9253) and 4.0% fewer surgeries for congenital heart disease, 0.6% (23,340 vs 23,205) more and 36.5% more surgeries for valvular heart disease, 5.9% (12,693 vs 13,445) and 42.3% fewer surgeries for ischemic heart procedures, and 5.0% (22,708 vs. 21,624) and 89.9% more surgeries for thoracic aortic aneurysm, respectively. Data for individual categories are summarized in Tables 1, 2, 3, 4, 5, 6.

**Table 1:** Congenital (total; 9006)

(1) CPB (+) (total; 6890)																				
	Neonate				Infant				1–17 years				≥ 18 years				Total			
	Cases	30-day mortality		Hospital mortality	Cases	30-day mortality		Hospital mortality	Cases	30-day mortality		Hospital mortality	Cases	30-day mortality		Hospital mortality	Cases	30-day mortality		Hospital mortality
		Hospital	After discharge			Hospital	After discharge			Hospital	After discharge			Hospital	After discharge			Hospital	After discharge	
PDA	5				4	1 (25.0)		1 (25.0)					24	1 (4.2)		1 (4.2)	33	2 (6.1)		2 (6.1)
Coarctation (simple)	10	1 (10.0)		1 (10.0)	13				15				13			1 (7.7)	51	1 (2.0)		2 (3.9)
+ VSD	48	1 (2.1)		1 (2.1)	44	1 (2.3)		1 (2.3)	13				0				105	2 (1.9)		2 (1.9)
+ DORV	3				4								0				7			
+ AVSD	1			1 (100.0)	2				1				0				4			1 (25.0)
+ TGA	0				2								0				2			
+ SV	1				0				1				0				2			
+ Others	7				4				3				0				14			
Interrupt. of Ao (simple)	0				0				0				0				0			
+ VSD	20				25	1 (4.0)		1 (4.0)	12				0				57	1 (1.8)		1 (1.8)
+ DORV	0				0				0				0				0			
+ Truncus	4	1 (25.0)		1 (25.0)	7			1 (14.3)	2				0				13	1 (7.7)		2 (15.4)
+ TGA	0				0				0				0				0			
+ Others	1				2				2				1				6			
Vascular ring	0				1				0				0				1			
PS	1				22				62	1 (1.6)		1 (1.6)	26	1 (3.8)		1 (3.8)	111	2 (1.8)		2 (1.8)
PA・IVS or Critical PS	10		1 (10.0)	1 (10.0)	52	1 (1.9)		2 (3.8)	65				9				136	1 (0.7)	1 (0.7)	3 (2.2)
TAPVR	106	7 (6.6)		11 (10.4)	49	1 (2.0)		1 (2.0)	20				0				175	8 (4.6)		12 (6.9)
PAPVR ± ASD	1				6				46				23				76			
ASD	1				49				526				873	6 (0.7)		6 (0.7)	1,449	6 (0.4)		6 (0.4)
Cor triatriatum	1				10				8				1				20			
AVSD (partial)	2				10			1 (10.0)	32				8				52			1 (1.9)
AVSD (complete)	6				102	1 (1.0)		1 (1.0)	110			2 (1.8)	4				222	1 (0.5)		3(1.4)
+ TOF or DORV	0				8				18				3				29			
+ Others	0				0				0				0				0			
VSD (subarterial)	1				85				156				7				249			
VSD (perimemb./muscular)	14				663			2 (0.3)	372			2 (0.5)	23				1,072			4 (0.4)
VSD (Type Unknown)	0				1				1				132	2 (1.5)		2 (1.5)	134	2 (1.5)		2 (1.5)
VSD + PS	1				24				12				3				40			
DCRV ± VSD	1				5				33				24				63			
Aneurysm of sinus of Valsalva	0								1				2				3			
TOF	11				172	1 (0.6)		1 (0.6)	184			2 (1.1)	35				402	1 (0.2)		3 (0.7)
PA + VSD	5				56				115	2 (1.7)		2 (1.7)	9				185	2 (1.1)		2 (1.1)
DORV	28	1 (3.6)		1 (3.6)	122			2 (1.6)	160	1 (0.6)		1 (0.6)	9				319	2 (0.6)		4 (1.3)
TGA (simple)	92	2 (2.2)		2 (2.2)	5				3				3				103	2 (1.9)		2 (1.9)
+ VSD	23			1 (4.3)	19				13				2				57			1 (1.8)
VSD + PS	0								2				0				2			
Corrected TGA	3				9	1 (11.1)		1 (11.1)	37				4				53	1 (1.9)		1 (1.9)
Truncus arteriosus	5			1 (20.0)	16			1 (6.3)	24				3				48			2 (4.2)
SV	28	2 (7.1)		4 (14.3)	143	4 (2.8)		4 (2.8)	160	2 (1.3)		3 (1.9)	16			1 (6.3)	347	8 (2.3)		12 (3.5)
TA	5			1 (20.0)	34				38				9				86			1 (1.2)
HLHS	28			7 (25.0)	114	1 (0.9)		8 (7.0)	72	1 (1.4)		1 (1.4)	1				215	2 (0.9)		16 (7.4)
Aortic valve lesion	3				25		1 (4.0)		115	1 (0.9)		1 (0.9)	42	1 (2.4)		1 (2.4)	185	2 (1.1)	1 (0.5)	2 (1.1)
Mitral valve lesion	0				31	1 (3.2)		1 (3.2)	70			1 (1.4)	26				127	1 (0.8)		2 (1.6)
Ebstein	14			2 (14.3)	13			1 (7.7)	19				17				63			3 (4.8)
Coronary disease	2				9	1 (11.1)		2 (22.2)	18				4				33	1 (3.0)		2 (6.1)
Others	10	1 (10.0)		1 (10.0)	30	2 (6.7)		4 (13.3)	39				191	4 (2.1)		4 (2.1)	270	7 (2.6)		9 (3.3)
Conduit failure	0				0				16				5				21			
Redo (excluding conduit failure)	3				56	2 (3.6)		6 (10.7)	100	2 (2.0)		5 (5.0)	89	1 (1.1)		2 (2.2)	248	5 (2.0)		13 (5.2)
Total	505	16 (3.2)	1 (0.2)	36 (7.1)	2,048	19 (0.9)	1 (0.05)	42 (2.1)	2,696	10 (0.4)	0	21 (0.8)	1,641	16 (1.0)	0	19 (1.2)	6,890	61 (0.9)	2 (0.0)	118 (1.7)
( ), % mortality																				
*CPB* cardiopulmonary bypass; *PDA* patent ductus arteriosus; *VSD* ventricular septal defect; *DORV* double outlet right ventricle; *AVSD* atrioventricular septal defect; *TGA* transposition of great arteries; *SV* single ventricle; *Interrupt. of Ao.* interruption of aortá; *PS* pulmonary stenosis; *PA-IVS* pulmonary atresia with intact ventricular septum; *TAPVR* total anomalous pulmonary venous return; *PAPVR* partial anomalous pulmonary venous return; *ASD* atrial septal defect; *TOF* tetralogy of Fallot; *DCRV* double-chambered right ventricle; *TA* tricuspid atresia; *HLHS* hypoplastic left heart syndrome; *RV-PA* right ventricle-pulmonary artery																				

(2) CPB (−) (total; 2116)																				
	Neonate				Infant				1-17 years				≥ 18 years				Total			
	Cases	30-day mortality		Hospital mortality	Cases	30-day mortality		Hospital mortality	Cases	30-day mortality		Hospital mortality	Cases	30-day mortality		Hospital mortality	Cases	30-day mortality		Hospital mortality
		Hospital	After discharge			Hospital	After discharge			Hospital	After discharge			Hospital	After discharge			Hospital	After discharge	
PDA	246	5 (2.0)		11 (4.5)	137	1 (0.7)		3 (2.2)	13				0				396	6 (1.5)		14 (3.5)
Coarctation (simple)	12				16				2				1				31			
+ VSD	48			3 (6.3)	19	1 (5.3)	1 (5.3)	2 (10.5)	3				0				70	1 (1.4)	1 (1.4)	5 (7.1)
+ DORV	3				0				0				0				3			
+ AVSD	2				0				0				0				2			
+ TGA	2				1				0				0				3			
+ SV	0				0				0				0				0			
+ Others	5				5				0				1				11			
Interrupt. of Ao (simple)	0				0				0				0				0			
+ VSD	20	1 (5.0)		1 (5.0)	9				1				0				30	1 (3.3)		1 (3.3)
+ DORV	0				0				1				0				1			
+ Truncus	8	1 (12.5)		1 (12.5)	0				0				0				8	1 (12.5)		1 (12.5)
+ TGA	0				0				0				0				0			
+ Others	3	1 (33.3)		1 (33.3)	1				0				0				4	1 (25.0)		1 (25.0)
Vascular ring	5				17				10				1				33			
PS	1				3				0				0				4			
PA・IVS or Critical PS	14				19	1 (5.3)		2 (10.5)	10				0				43	1 (2.3)		2 (4.7)
TAPVR	16	5 (31.3)		6 (37.5)	17	2 (11.8)		2 (11.8)	1				0				34	7 (20.6)		8 (23.5)
PAPVR ± ASD	0				0				1				0				1			
ASD	1				2				2				4				9			
Cor triatriatum	0				0				0				0				0			
AVSD (partial)	1				0				3				0				4			
AVSD (complete)	34				81		1 (1.2)		9			1 (11.1)	2				126		1 (0.8)	1 (0.8)
+ TOF or DORV	1				3				2				0				6			
+ Others	0				0				0				0				0			
VSD (subarterial)	1				6				2				0				9			
VSD (perimemb./muscular)	56	1 (1.8)		2 (3.6)	127	1 (0.8)		2 (1.6)	13			1 (7.7)	0				196	2 (1.0)		5 (2.6)
VSD (Type Unknown)	0								0				2				2			
VSD + PS	0								0				0				0			
DCRV ± VSD	0								0				0				0			
Aneurysm of sinus of Valsalva	0				0				0				0				0			
TOF	13				62				19	1 (5.3)		1 (5.3)	3				97	1 (1.0)		1 (1.0)
PA + VSD	12				40				12				2				66			
DORV	41	2 (4.9)		2 (4.9)	61			1 (1.6)	14			1 (7.1)	2				118	2 (1.7)		4 (3.4)
TGA (simple)	4				4	1 (25.0)		1 (25.0)	1				2				11	1 (9.1)		1 (9.1)
+ VSD	7				2				0				1				10			
VSD + PS	0				0				0				0				0			
Corrected TGA	5			1 (20.0)	6				7				1				19			1 (5.3)
Truncus arteriosus	24			1 (4.2)	1				1				0				26			1 (3.8)
SV	60	1 (1.7)		3 (5.0)	40	1 (2.5)		3 (7.5)	18	2 (11.1)		2 (11.1)	6				124	4 (3.2)		8 (6.5)
TA	13				7				0				1				21			
HLHS	85	2 (2.4)		10 (11.8)	31	3 (9.7)		5 (16.1)	23	2 (8.7)		2 (8.7)	0				139	7 (5.0)		17 (12.2)
Aortic valve lesion	4			1 (25.0)	3		1 (33.3)		0				0				7		1 (14.3)	1 (14.3)
Mitral valve lesion	3				2	1 (50.0)		1 (50.0)	1				1				7	1 (14.3)		1 (14.3)
Ebstein	10	1 (10.0)		2 (20.0)	3				5				0				18	1 (5.6)		2 (11.1)
Coronary disease	0				6	1 (16.7)		2 (33.3)	2				2				10	1 (10.0)		2 (20.0)
Others	6				11				17	3 (17.6)		4 (23.5)	2				36	3 (8.3)		4 (11.1)
Conduit failure	0								0				0				0			
Redo (excluding conduit failure)	37	9 (24.3)		13 (35.1)	154	9 (5.8)		24 (15.6)	165	7 (4.2)		14 (8.5)	25				381	25 (6.6)		51 (13.4)
Total	803	29 (3.6)	0	58 (7.2)	896	22 (2.5)	3 (0.3)	48 (5.4)	358	15 (4.2)	0	26 (7.3)	59	0	0	0	2,116	66 (3.1)	3 (0.14)	132 (6.2)
( ), % mortality																				
*CPB* cardiopulmonary bypass; *PDA* patent ductus arteriosus; *VSD* ventricular septal defect; *DORV* double outlet right ventricle; *AVSD* atrioventricular septal defect; *TGA* transposition of the great arteries; *SV* single ventricle; *Interrupt. of Ao.* interruption of aorta; *PS* pulmonary stenosis; *PA-IVS* pulmonary atresia with intact ventricular septum; *TAPVR* total anomalous pulmonary venous return; *PAPVR* partial anomalous pulmonary venous return; *ASD* atrial septal defect; *TOF* tetralogy of Fallot; *DCRV* double-chambered right ventricle; *TA* tricuspid atresia; *HLHS* hypoplastic left heart syndrome; *RV-PA* right ventricle-pulmonary artery																				

(3) Main procedure																					
		Neonate				Infant				1- 17 years				≥ 18 years				Total			
		Cases	30-day mortality		Hospital mortality	Cases	30-day mortality		Hospital mortality	Cases	30-day mortality		Hospital mortality	Cases	30-day mortality		Hospital mortality	Cases	30-day mortality		Hospital mortality
			Hospital	After discharge			Hospital	After discharge			Hospital	After discharge			Hospital	After discharge			Hospital	After discharge	
1	SP Shunt	96	2 (2.1)		4 (4.2)	330	4 (1.2)		9 (2.7)	51	1 (2.0)		2 (3.9)	4				481	7 (1.5)		15 (3.1)
2	PAB	293	8 (2.7)		19 (6.5)	306	3 (1.0)	1 (0.3)	8 (2.6)	15				2				616	11 (1.8)	1 (0.2)	27 (4.4)
3	Bidirectional Glenn or hemi-Fontan ±α	0				215	4 (1.9)		6 (2.8)	109				5				329	4 (1.2)		6 (1.8)
4	Damus-Kaye-Stansel operation	3					28	1 (3.6)		1 (3.6)	12				1				44	1 (2.3)	1 (2.3)
5	PA reconstruction/repair (including redo)	12	1 (8.3)	1 (8.3)	2 (16.7)	164	1 (0.6)		4 (2.4)	177	1 (0.6)		2 (1.1)	31				384	3 (0.8)	1 (0.3)	8 (2.1)
6	RVOT reconstruction/repair	6				206	1 (0.5)		2 (1.0)	281	1 (0.4)		2 (0.7)	42				535	2 (0.4)		4 (0.7)
7	Rastelli procedure	0				33				109			1 (0.9)	5				147			1 (0.7)
8	Arterial switch procedure	130	3 (2.3)		5 (3.8)	24				4				1				159	3 (1.9)		5 (3.1)
9	Atrial switch procedure	1				2			1 (50.0)	4				1				8			1 (12.5)
10	Double switch procedure	0				1				7				0				8			
11	Repair of anomalous origin of CA	0				8	1 (12.5)		2 (25.0)	5				0				13	1 (7.7)		2 (15.4)
12	Closure of coronary AV fistula	3				2				2				4				11			
13	Fontan / TCPC	0				1				360	2 (0.6)		4 (1.1)	34	1 (2.9)	1 (2.9)	2 (5.9)	395	3 (0.8)	1 (0.3)	6 (1.5)
14	Norwood procedure	25			3 (12.0)	88	1 (1.1)		6 (6.8)	3				0				116	1 (0.9)		9 (7.8)
15	Ventricular septation	0				0				0				0				0			
16	Left side AV valve repair (including Redo)	1				38				73				22				134			
17	Left side AV valve replace (including Redo)	0				12				45			1 (2.2)	22			2 (9.1)	79			3 (3.8)
18	Right side AV valve repair (including Redo)	24			3 (12.5)	77	2 (2.6)		6 (7.8)	91	1 (1.1)		1 (1.1)	81			1 (1.2)	273	3 (1.1)		11 (4.0)
19	Right side AV valve replace (including Redo)	1			1 (100.0)	1				13			1 (7.7)	22				37			2 (5.4)
20	Common AV valve repair (including Redo)	9	2 (22.2)		2 (22.2)	8	2 (25.0)		2 (25.0)	25	1 (4.0)		1 (4.0)	1				43	5 (11.6)		5 (11.6)
21	Common AV valve replace (including Redo)	0				5				11		1 (9.1)	1 (9.1)	1		1 (100.0)		17		2 (11.8)	1 (5.9)
22	Repair of supra-aortic stenosis	0				9	1 (11.1)	1 (11.1)	1 (11.1)	18				2				29	1 (3.4)	1 (3.4)	1 (3.4)
23	Repair of subaortic stenosis (including Redo)	0				1				35				6				42			
24	Aortic valve plasty ± VSD Closure	5				16				48	1 (2.1)		1 (2.1)	1				70	1 (1.4)		1 (1.4)
25	Aortic valve replacement	0				0				32	1 (3.1)		1 (3.1)	43	1 (2.3)	2 (4.7)	1 (2.3)	75	2 (2.7)	2 (2.7)	2 (2.7)
26	AVR with annular enlargement	0				0				13				3		1 (33.3)		16		1 (6.3)	
27	Aortic root Replace (except Ross)	0				0				6			1 (16.7)	18	1 (5.6)	1 (5.6)		24	1 (4.2)	1 (4.2)	1 (4.2)
28	Ross procedure	0				5				14								19			
29	Bilateral pulmonary artery banding	160	2 (1.3)		15 (9.4)	12	1 (8.3)		2 (16.7)	1				0				173	3 (1.7)		17 (9.8)
Total		769	18 (2.3)	1 (0.1)	54 (7.0)	1,592	22 (1.4)	2 (0.1)	50 (3.1)	1,564	9 (0.6)	1 (0.1)	19 (1.2)	352	3 (0.9)	6 (1.7)	6 (1.7)	4,277	52 (1.2)	10 (0.23)	129 (3.0)

( ), % mortality

*SP* systemic-pulmonary; *PAB* pulmonary artery banding; *PA* pulmonary artery; *RVOT* right ventricular outflow tract; *CA* coronary artery; *AV fistula* arteriovenous fistula; *TCPC* total cavopulmonary connection; *AV valve* atrioventricular valve; *VSD* ventricular septal defect; *AVR* aortic valve replacement

**Table 2:** Acquired (total, (1) + (2) + (4) + (5) + (6) + (7) + isolated operations for arrhythmia in (3); 38,592

(1) Valvular heart disease (total; 23,340)																	
	Valve	Cases	Operation					30-Day mortality				Hospital mortality		Redo			
			Mechanical	Bioprosthesis	Repair	Unknown	With CABG	Hospital		After discharge				Cases	30-Day mortality		Hospital mortality
								Replace	Repair	Replace	Repair	Replace	Repair		Hosipital	After discharge	
Isolated	A	10,268	1,271	8,720	178	99	2489	175 (1.8)	4 (2.3)	2 (0.02)	0	294 (2.9)	5 (2.8)	670	31 (4.6)	0	45 (6.7)
	M	5,239	428	970	3,810	31	560	56 (4.0)	30 (0.8)	2 (0.1)	0	89 (6.4)	47 (1.2)	620	25 (4.0)	0	38 (6.1)
	T	629	6	107	511	5	58	2 (1.8)	18 (3.5)	0	0	7 (6.2)	32 (6.3)	122	7 (5.7)	0	15 (12.3)
	P	31	0	26	5	0	0	0	0	0	0	0	0	19	0	0	0
A+M		1,345					202	54 (4.0)		0		88 (6.5)		173	12 (6.9)	0	21 (12.1)
	A		238	1057	42	8											
	M		160	463	714	8											
A+T		564					94	10 (1.8)		0		30 (5.3)		77	2 (2.6)	0	6 (7.8)
	A		61	485	11	7											
	T		2	12	547	3											
M+T		4,033					327	58 (1.8)		1 (0.02)		99 (2.5)		466	16 (3.4)	0	23 (4.9)
	M		378	1,118	2,519	18											
	T		1	51	3,961	20											
A+M+T		1,143					122	42 (3.7)		0		70 (6.1)		111	5 (4.5)	0	12 (10.8)
	A		161	945	26	11											
	M		112	436	589	6											
	T		2	3	1,135	3											
Others		88					7	2 (2.3)		0		3 (3.4)		22	0	0	1 (4.6)
Unknown								4				6					
Total		23,340					3859	456 (2.0)		5 (0.02)		770 (3.3)		2,280	98 (4.3)	0	161 (7.1)
( ), % mortality																	

TAVR	Cases	30-day mortality
	8664	103 (1.2)

(2) Ischemic heart disease (total, (A) + (B) ; 12,603)																					
(A) Isolated CABG (total; (a)+(b); 11307)																					
(a-1) On-pump arrest CABG (total;2491)																					
	Primary, elective				Primary, emergent				Redo, elective				Redo, emergent				Artery only	Artery +svg	Svg only	Others	Unclear
	Cases	30 Day mortality		Hospital mortality	Cases	30 Day mortality		Hospital mortality	Cases	30 Day mortality		Hospital mortality	Cases	30 day mortality		Hospital mortality					
		Hospital	After discharge			Hospital	After discharge			Hospital	After discharge			Hospital	After discharge						
1VD	45				11				4				0				18	29	10	2	1
2VD	273	2 (0.7)		2 (0.7)	47	3 (6.4)		5 (10.6)	2				0				37	257	23	3	2
3VD	926	8 (0.9)	1 (0.1)	13 (1.4)	130	9 (6.9)		12 (9.2)	2				0				45	946	47	6	14
LMT	781	10 (1.3)		20 (2.6)	240	14 (5.8)		22 (9.2)	5				0				60	890	60	9	7
No info	16	0			8			2 (25.0)	1				1	1 (100.0)		1 (100.0)	6	7	10	1	2
Total	2041	20 (1.0)	1 (0.0)	35 (1.7)	436	26 (6.0)		41 (9.4)	14				1	1 (100.0)		1 (100.0)	166	2129	150	21	26
Kawasaki	2				1				0				0				1	2	0	0	0
On dialysis	237	5 (2.1)	1 (0.4)	11 (4.6)	49	4 (8.2)		9 (18.4)	1				0				11	248	25	2	1
( ), % mortality																					
LMT includes LMT alone or LMT with other branch diseases																					
*CABG* coronary artery bypass grafting; *1VD* one-vessel disease; *2VD* two-vessel disease; *3VD* three-vessel disease; *LMT* left main trunk; *SVG* saphenous vein graft																					

(a-2) On-pump beating CABG (total;2,307)																					
	Primary, elective				Primary, emergent				Redo, elective				Redo, emergent				Artery only	Artery +svg	Svg only	Others	Unclear
	Cases	30 day Mortality		Hospital mortality	Cases	30 day mortality		Hospital mortality	Cases	30 day mortality		Hospital mortality	Cases	30 day mortality		Hospital mortality					
		Hospital	After discharge			Hospital	After discharge			Hospital	After discharge			Hospital	After discharge						
1VD	28			0 (0.0)	10	1 (10.0)		2 (20.0)	3			1	1	1 (100.0)		1 (100.0)	14	19	8	0	1
2VD	211	1 (0.5)		1 (0.5)	49	10 (20.4)		14 (28.6)	2				0				46	186	24	1	5
3VD	765	14 (1.8)	2 (0.3)	24 (3.1)	187	13 (7.0)		22 (11.8)	9	1 (11.1)		1 (11.1)	1	1 (100.0)		1 (100.0)	72	841	36	9	4
LMT	676	5 (0.7)		14 (2.1)	330	14 (4.2)		27 (8.2)	10			1 (10.0)	2	2 (100.0)		2 (100.0)	127	834	50	2	5
no info	16	1 (6.3)		1 (6.3)	11	1 (9.1)		1 (9.1)	0				4	1 (25.0)		1 (25.0)	8	15	7	1	0
Total	1696	21 (1.2)	2 (0.1)	40 (2.4)	587	39 (6.6)		66 (11.2)	24	1 (4.2)		3 (12.5)	8	5 (62.5)		5 (62.5)	267	1895	125	13	15
Kawasaki	1				0				0				0				0	1	0	0	0
On dialysis	214	12 (5.6)		19 (8.9)	80	7 (8.8)		16 (20.0)	5	1 (20.0)		2 (40.0)	1	1 (100.0)		1 (100.0)	22	253	22	1	2
( ), % mortality																					
LMT includes LMT alone or LMT with other branch diseases																					
*CABG* coronary artery bypass grafting; *1VD* one-vessel disease; *2VD* two-vessel disease; *3VD* three-vessel disease; *LMT* left main trunk; *SVG* saphenous vein graft																					

(b) Off-pump CABG (total;6509)																					
(Including cases of planned off-pump CABG in which, during surgery, the change is made to an on-pump CABG or on-pump beating-heart procedure)																					
	Primary, elective				Primary, emergent				Redo, elective				Redo, emergent				Artery only	Artery +svg	Svg only	Others	Unclear
	Cases	30 day mortality		Hospital mortality	Cases	30 day mortality		Hospital mortality	Cases	30 day mortality		Hospital mortality	Cases	30 Day mortality		Hospital mortality					
		Hospital	After discharge			Hospital	After discharge			Hospital	After discharge			Hospital	After discharge						
1VD	352	2 (0.6)		2 (0.6)	44	3 (6.8)		5 (11.4)	2				3	1 (33.3)		1 (33.3)	293	71	35	1	1
2VD	860	3 (0.3)		13 (1.5)	112	3 (2.7)	1 (0.9)	4 (3.6)	7	1 (14.3)		1 (14.3)	1	1 (100.0)		1 (100.0)	335	606	33	0	6
3VD	2158	15 (0.7)		26 (1.2)	305	6 (2.0)		14 (4.6)	12	1 (8.3)		1 (8.3)	1				468	1931	50	11	16
LMT	2028	17 (0.8)	1 (0.0)	30 (1.5)	513	23 (4.5)	1 (0.2)	30 (5.8)	18	1 (5.6)		1 (5.6)	9	1 (11.1)		1 (11.1)	667	1801	86	5	9
No info	83	0 (0.0)		0 (0.0)	13	1 (7.7)		1 (7.7)	2				2	1 (50.0)		1 (50.0)	24	63	9	1	3
Total	5481	37 (0.7)	1 (0.0)	71 (1.3)	987	36 (3.6)	2 (0.2)	54 (5.5)	41	3 (7.3)		3 (7.3)	16	4 (25.0)		4 (25.0)	1787	4472	213	18	35
Kawasaki	0							0	0				0	0			0	0	0	0	0
On dialysis	556	8 (1.4)		22 (4.0)	90	4 (4.4)		6 (6.7)	7	1 (14.3)		1 (14.3)	5	2 (40.0)		2 (40.0)	174	448	29	2	5
( ), % mortality																					
LMT includes LMT alone or LMT with other branch diseases																					
*CABG* coronary artery bypass grafting; *1VD* one-vessel disease; *2VD* two-vessel disease; *3VD* three-vessel disease; *LMT* left main trunk; *SVG* saphenous vein graft																					

(c) Cases of conversion, during surgery, from off-pump CABG to on-pump CABG or on- pump beating-heart CABG (these cases are also included in category (b))																
	Primary, elective				Primary, emergent				Redo, elective				Redo, emergent			
	Cases	30 Day mortality		Hospital mortality	Cases	30 Day mortality		Hospital mortality	Cases	30 Day mortality		Hospital mortality	Cases	30 Day mortality		Hossspital mortality
		Hospital	After discharge			Hospital	After discharge			Hospital	After discharge			Hospital	After discharge	
Converted to arrest	21			2 (9.5)	3								0			
Converted to beating	102	6 (5.9)		9 (8.8)	39	3 (7.7)		7 (17.9)	6	1 (16.7)		1 (16.7)	1	1 (100.0)		1 (100.0)
Total	123	6 (4.9)		11 (8.9)	42	3 (7.1)		7 (16.7)	6	1 (16.7)		1 (16.7)	1	1 (100.0)		1 (100.0)
On dialysis	21	2 (9.5)		5 (23.8)	7	1 (14.3)		3 (42.9)	5	1 (20.0)		1 (20.0)	0			
( ), % mortality																
*CABG* coronary artery bypass grafting																

(B) Operation for complications of MI (total; 1296)											
	Chronic				Acute				Concomitant operation		
	Cases	30-day mortality		Hospital mortality	Cases	30-day mortality		Hospital mortality	CABG	MVP	MVR
		Hospital	After discharge			Hospital	After discharge				
Infarctectomy or Aneurysmectomy	99	6 (6.1)		8 (8.1)	24	8 (33.3)		9 (37.5)	55	25	8
VSP closure	81	9 (11.1)		13 (16.0)	262	68 (26.0)		102 (38.9)	90	4	6
Cardiac rupture	29	7 (24.1)		11 (37.9)	238	78 (32.8)		90 (37.8)	36	2	5
Mitral regurgitation											
(1) Papillary muscle rupture	74	4 (5.4)		4 (5.4)	52	14 (26.9)		19 (36.5)	20	10	56
(2) Ischemic	216	15 (6.9)		25 (11.6)	42	9 (21.4)		11 (26.2)	171	151	107
Others	78	7 (9.0)		10 (12.8)	101	22 (21.8)		35 (34.7)	72	9	7
Total	577	48 (8.3)		71 (12.3)	719	199 (27.7)		266 (37.0)	444	201	189
( ), % mortality											
*MI* myocardial infarction; *CABG* coronary artery bypass grafting; *MVP* mitral valve repair; *MVR* mitral valve replacement; *VSP* ventricular septal perforation											
Acute, within 2 weeks from the onset of myocardial infarction											

(3) Operation for arrhythmia (total;6880 )											
	Cases	30-day mortality		Hospital mortality	Concomitant operation						
					Isolated	Congenital	Valve	IHD	Others	Multiple combination	
		Hospital	After discharge							2 categories	3 categories
Maze	3,898	66 (1.7)	1 (0.03)	119 (3.1)	157	169	3,345	636	364	699	61
For WPW	0				0	0	0	0	0	0	0
For ventricular tachyarrhythmia	32	2 (6.3)		3 (9.4)	5	3	12	18	1	8	1
Others	2,950	57 (1.9)		96 (3.3)	85	129	2,500	525	285	533	48
Total	6,880	125 (1.8)	1 (0.01)	218 (3.2)	247	301	5,857	1179	650	1,240	110
( ), % mortality											
*WPW* Wolff-Parkinson-White syndrome; *IHD* ischemic heart disease											
Except for 247 isolated cases, all remaining 6633 cases are doubly allocated, one for this subgroup and the other for the subgroup corresponding to the concomitant operations											

(4) Operation for constrictive pericarditis (total; 191)								
	CPB (+)				CPB (-)			
	Cases	30-day mortality		Hospital mortality	Cases	30-day mortality		Hospital mortality
		Hospital	After discharge			Hospital	After discharge	
Total	95	4 (4.2)		18 (18.9)	96		1 (1.0)	3 (3.1)
( ), % mortality								
*CPB* cardiopulmonary bypass								

(5) Cardiac tumor (total; 704)								
	Cases	30-day mortality		Hospital mortality	Concomitant operation			
		Hospital	After discharge		AVR	MVR	CABG	Others
Benign tumor	640	3 (0.5)			32	34	45	143
(Cardiac myxoma)	393	1 (0.3)			13	3	24	72
Malignant tumor	64	2 (3.1)		6 (9.4)	2	4	4	11
(Primary)	43	1 (2.3)		3 (7.0)	2	4	4	10
( ), % mortality								
*AVR* aortic valve replacement; *MVR* mitral valve replacement; *CABG* coronary artery bypass grafting								

(6) HOCM and DCM (total; 278)								
	Cases	30-day mortality		Hospital mortality	Concomitant operation			
		Hospital	After discharge		AVR	MVR	MVP	CABG
Myectomy	130	5 (3.8)		7 (5.4)	56	26	21	21
Myotomy	13				1	1	1	1
No-resection	128	10 (7.8)	1 (0.8)	1 (0.8)	21	74	54	6
Volume reduction surgery of the left ventricle	7		1 (0.4)		1	3	1	2
Total	278	15 (5.4)		8 (2.9)	79	104	77	30
( ), % mortality								
*HOCM* hypertrophic obstructive cardiomyopathy; *DCM* dilated cardiomyopathy; *AVR* aortic valve replacement; *MVR* mitral valve replacement; *MVP* mitral valve repair; *CABG* coronary artery bypass grafting								

(7) Other open-heart operation (total; 1229)				
	Cases	30-day mortality		Hospital mortality
		Hospital	After discharge	
Open-heart operation	523	52 (9.9)	1 (0.2)	75 (14.3)
Non-open-heart operation	706	88 (12.5)		120 (17.0)
Total	1229	140 (11.4)	1 (0.1)	195 (15.9)

( ), % mortality

**Table 3:** Thoracic aortic aneurysm (total; 22,708) (1) Dissection (total; 10,847)

Stanford type	Acute								Chronic								Concomitant operation					
	A				B				A				B									
Replaced site	Cases	30-day mortality		Hospital mortality	Cases	30-day mortality		Hospital mortality	Cases	30-day mortality		Hospital mortality	Cases	30-day mortality		Hospital mortality	AVP	AVR	MVP	MVR	CABG	Others
		Hospital	After discharge			Hospital	After discharge			Hospital	After discharge			Hospital	After discharge							
Ascending Ao.	2376	177 (7.4)	0	235 (9.9)	2				211	8 (3.8)		12 (5.7)	6	1 (16.7)		1 (16.7)	74	139	13	19	135	30
Aortic Root	232	29 (12.5)	3 (1.29)	36 (15.5)	0				92	7 (7.6)		11 (12.0)	5				47	206	4	2	72	3
Arch	2045	144 (7.0)	2 (0.10)	175 (8.6)	23	2 (8.7)		2 (8.7)	353	6 (1.7)		9 (2.5)	166	5 (3.0)		5 (3.0)	63	141	10	10	130	27
Aortic root + asc. Ao. + Arch	173	20 (11.6)	0	26 (15.0)	1				51	3 (5.9)		4 (7.8)	8	2 (25.0)		3 (37.5)	37	149	2	1	53	3
Descending Ao.	43	3 (7.0)	0	3 (7.0)	42	4 (9.5)		5 (11.9)	80	1 (1.3)		4 (5.0)	249	12 (4.8)		17 (6.8)	4	5	0	0	6	0
Thoracoabdominal	2	0	0	0	11	2 (18.2)		2 (18.2)	49	3 (6.1)		5 (10.2)	171	10 (5.8)		16 (9.4)	0	0	0	0	0	0
Simple TEVAR	69	14 (20.3)	0	16 (23.2)	412	22 (5.3)		32 (7.8)	233	2 (0.9)		4 (1.7)	1067	15 (1.4)	1 (0.1)	20 (1.9)	0	0	0	0	1	2
Open SG with BR	993	75 (7.6)	0	106 (10.7)	52	7 (13.5)		12 (23.1)	191	4 (2.1)		8 (4.2)	193	4 (2.1)		5 (2.6)	49	99	3	1	90	10
Open SG without BR	370	36 (9.7)	1 (0.27)	52 (14.1)	29	3 (10.3)		5 (17.2)	67	5 (7.5)		6 (9.0)	74	2 (2.7)		4 (5.4)	18	44	4	1	36	4
Arch TEVAR with BR	18	2 (11.1)	0	2 (11.1)	108	7 (6.5)	1 (0.9)	9 (8.3)	57				374	5 (1.3)		7 (1.9)	0	1	0	0	0	0
Thoracoabdominal TEVAR with BR	2	0	0	0	6	1 (16.7)		1 (16.7)	12				29	2 (6.9)		4 (13.8)	0	1	0	0	0	0
Other	24	9 (37.5)	0	10 (41.7)	17	1 (5.9)		1 (5.9)	16	1 (6.3)		1 (6.3)	43				1	1	0	1	3	2
Total	6347	387 (6.1)	6 (0.09)	661 (10.4)	703	49 (7.0)	1 (0.1)	69 (9.8)	1412	40 (2.8)	0	64 (4.5)	2385	58 (2.4)	1 (0.0)	82 (3.4)	293	786	36	35	526	81
( ), % mortality																						
*Ao* aorta; *AVP* aortic valve repair; *AVR* aortic valve replacement; *MVP* mitral valve repair; *MVR* mitral valve replacement; *CABG* coronary artery bypass grafting; *TEVAR* thoracic endovascular aortic (aneurysm) repair																						
Acute, within 2 weeks from the onset																						

(2) Non-dissection (total; 11861)														
Replaced site	Unruptured				Ruptured				Concomitant operation					
	Cases	30-day mortality		Hospital mortality	Cases	30-day mortality		Hospital mortality	AVP	AVR	MVP	MVR	CABG	Others
		Hospital	After discharge			Hospital	After discharge							
Ascending Ao.	1440	22 (1.5)		45 (3.1)	60	13 (21.7)		14 (23.3)	79	1012	84	57	189	120
Aortic Root	1174	35 (3.0)		51 (4.3)	45	5 (11.1)		5 (11.1)	304	818	71	36	162	63
Arch	2243	38 (1.7)		76 (3.4)	103	10 (9.7)		16 (15.5)	39	589	41	24	302	68
Aortic root + asc. Ao. + Arch	286	9 (3.1)		12 (4.2)	8	1 (12.5)		1 (12.5)	45	214	13	5	33	14
Descending Ao.	344	16 (4.7)		20 (5.8)	35	11 (31.4)		15 (42.9)	1	7	2	0	21	1
Thoracoabdominal	356	24 (6.7)		34 (9.6)	27	6 (22.2)		8 (29.6)	0	0	0	0	0	0
Simple TEVAR	2496	46 (1.8)		67 (2.7)	340	37 (10.9)	3 (0.88)	56 (16.5)	0	0	0	0	1	8
Open SG with BR	1066	37 (3.5)		62 (5.8)	52	6 (11.5)		8 (15.4)	15	120	11	1	192	18
Open SG without BR	354	9 (2.5)		21 (5.9)	27	2 (7.4)		4 (14.8)	13	52	6	1	54	3
Arch TEVAR with BR	1042	33 (3.2)	1 (0.10)	57 (5.5)	85	12 (14.1)		18 (21.2)	0	1	0	1	6	0
Thoracoabdominal TEVAR with BR	95	3 (3.2)		12 (12.6)	14	6 (42.9)		8 (57.1)	0	0	0	0	0	0
Other	142	7 (4.9)		11 (7.7)	27	5 (18.5)		9 (33.3)	0	15	0	3	6	2
Total	11038	279 (2.5)	1 (0.01)	468 (4.2)	823	114 (13.9)	3 (0.36)	162 (19.7)	496	2828	228	128	966	297

( ), % mortality

*Ao* aorta; *AVP* aortic valve repair; *AVR* aortic valve replacement; *MVP* mitral valve repair; *MVR* mitral valve replacement; *CABG* coronary artery bypass grafting; *TEVAR* thoracic endovascular aortic (aneurysm) repair

Acute, within 2 weeks from the onset

**Table 4:** Pulmonary thromboembolism (total; 187)

	Cases	30-day mortality		Hospital mortality
		Hospital	After discharge	
Acute	125	20 (16.0)		22 (17.6)
Chronic	62	2 (3.2)		2 (3.2)
Total	187	22 (11.8)		24 (12.8)

( ), % mortality

**Table 5:** Implantation of VAD (total; 192)

	Cases	30-day mortality		Hospital mortality
		Hospital	After discharge	
Implantation of VAD	192	2 (1.0)		9 (4.7)

( ), % mortality

*VAD* ventricular assist devise

**Table 6:** Heart transplantation (total; 84)

	Cases	30-day mortality		Hospital mortality
		Hospital	After discharge	
Heart transplantation	84	1 (1.2)		2 (2.4)
Heart and lung transplantation	0			
Total	84	1 (1.2)		2 (2.4)

( ), % mortality

Among the 9006 procedures for congenital heart disease conducted in 2019, 6890 were open-heart surgeries, with an overall hospital mortality rate of 1.7%. The number of surgeries for neonates and infants in 2019 did not significantly differ compared to that in 2009; however, hospital mortality improved from 10.7% to 7.1% for neonates and from 3.7% to 2.1% for infants. In 2019, atrial septal defect was the most common disease (1449 cases) as previously reported, with patients aged ≥ 18 years accounting for 60.2% of atrial septal defect surgery. Ventricular septal defect (perimembranous/muscular), which had been the most common disease in 2015 and 2016, was the second most common disease (1072 cases).

Hospital mortality for complex congenital heart disease within the past 10 years was as follows (2009 [2], 2014 [3], and 2019): complete atrioventricular septal defect (4.3%, 1.7%, and 1.4%); tetralogy of Fallot (1.8%, 1.1%, and 0.7%); transposition of the great arteries with the intact septum (4.2%, 6.6%, and 1.9%), ventricular septal defect (6.5%, 3.9%, and 1.8%), and single ventricle (4.3%, 4.3%, and 3.5%); and hypoplastic left heart syndrome (16.5%, 9.8%, and 7.4%). Currently, right heart bypass surgery has been commonly performed (329 bidirectional Glenn procedures, excluding 44 Damus–Kaye–Stansel procedures, and 395 Fontan type procedures, including total cavopulmonary connection) with acceptable hospital mortality rates (1.8% and 1.5%). The Norwood type I procedure was performed in 116 cases, with a relatively low hospital mortality rate (7.8%).

Valvular heart disease procedures, excluding transcatheter procedures, were slightly performed more than that in the previous year. Moreover, isolated aortic valve replacement/repair with/without coronary artery bypass grafting (CABG) (*n* = 10,268) was 3.0% lower than that in the previous year (*n* = 10,584) but 0.5% higher than that 5 years ago (*n* = 10,219), despite the rapid utilization of transcatheter aortic valve replacement (*n* = 8664 in 2019). Isolated mitral valve replacement/repairs with/without CABG (*n* = 5239) was 7.0% higher than that in the previous year (*n* = 4898) and 8.0% higher than that 5 years ago (*n* = 4851). Aortic and mitral valve replacement with bioprosthesis were performed in 11,207 and 2987 cases, respectively. The rate at which bioprosthesis was used had dramatically increased from 30% in the early 2000s [4, 5] to 83.0% and 73.0% in 2019 for aortic and mitral positions, respectively. Additionally, CABG was performed concurrently in 16.5% of all valvular procedures (17.2% in 2009 [2] and 17.3% in 2014 [3]). Valve repair was common in mitral and tricuspid valve positions (7632 and 6154 cases, respectively) but less common in aortic valve positions (257 patients, only 1.9% of all aortic valve procedures). Mitral valve repair accounted for 70.9% of all mitral valve procedures. Hospital mortality rates for single valve replacement for aortic and mitral positions were 2.9% and 6.4%, respectively, but only 1.2% for mitral valve repair. Moreover, hospital mortality rates for redo valve surgery for the aortic and mitral positions were 6.7% and 6.1%, respectively. Finally, overall hospital mortality rates did not significantly improve over the past 10 years (4.0% in 2009 [2], 3.1% in 2014 [3], and 3.3% in 2019).

Isolated CABG had been performed in 11,307 cases, accounting for only 68.3% of the procedures performed 10 years ago (*n* = 16,536) [2]. Of the aforementioned cases, 6509 (57.6%) underwent off-pump CABG, with a success rate of 97.8%. The percentage of planned off-pump CABG in 2019 was similar to that in 2018 when it fell below 60% for the first time since 2004 [4]. Hospital mortality associated with primary elective CABG procedures among 9218 cases accounted for 1.6%, which is slightly higher than that in 2009 (1.2%) [2]. Hospital mortality for primary emergency CABG among 1667 cases remained high (8.0%). The percentage of conversion from off-pump to on-pump CABG or on-pump beating-heart CABG was 2.2% among the primary elective CABG cases, with a hospital mortality rate of 8.9%. Patients with end-stage renal failure on dialysis had higher hospital mortality rates than overall mortality, regardless of surgical procedure (on-pump arrest, on-pump beating, and off-pump). This study excluded concomitant CABGs alongside other major procedures under the ischemic heart disease category but rather under other categories, such as valvular heart disease and thoracic aortic aneurysm. Accordingly, the overall number of CABGs in 2019, including concomitant CABG with other major procedures, was 17,256.

Arrhythmia management was primarily performed as concomitant procedures in 6880 cases, with a hospital mortality rate of 3.2%. Pacemaker and implantable cardioverter-defibrillator implantation were not included in this category.

In 2019, 22,708 procedures for thoracic and thoracoabdominal aortae diseases were performed, among which aortic dissection and non-dissection accounted for 10,847 and 11,861, respectively. The number of surgeries for aortic dissection this year was 3.8% higher than that in the preceding year (*n* = 10,453). Hospital mortality rates for the 6347 Stanford type A acute aortic dissections remained high (10.4%). The number of procedures for non-dissected aneurysms decreased by 1.2%, with a hospital mortality rate of 5.7% for all aneurysms and 4.2% and 19.7% for unruptured and ruptured aneurysms, respectively. Thoracic endovascular aortic repair (TEVAR) has been performed for aortic diseases at an increasing rate. Stent graft placement was performed in 4356 patients with aortic dissection, including 2387 TEVARs and 1969 open stent graftings. Moreover, 1470 and 267 cases underwent TEVAR and open stent grafting for type B chronic aortic dissection, accounting for 61.6% and 13.6% of the total number of cases, respectively. Hospital mortality rates associated with simple TEVAR for type B aortic dissection were 8.0% and 2.1% for acute and chronic cases, respectively. Stent graft placement was performed in 5087 patients with non-dissected aortic aneurysms, among which 4072 were TEVARs (an 11.8% increase compared to that in 2018, *n* = 3641) and 1499 were open stent graftings (a 3.7% increase compared to that in 2018, *n* = 1446). Hospital mortality rates were 3.7% and 18.7% for TEVARs and 5.8% and 15.2% for open stenting in unruptured and ruptured aneurysms, respectively.

### (B) General thoracic surgery

**Fig. 2:**
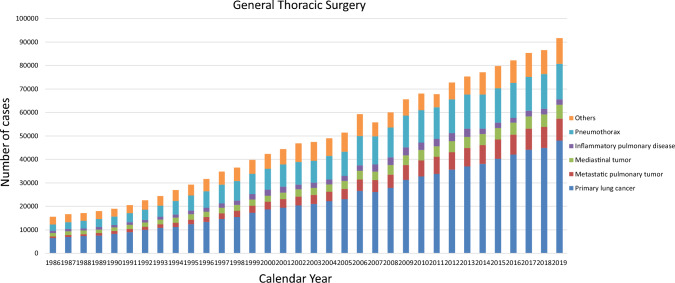
General thoracic surgery

The 2019 survey of general thoracic surgeries comprised 679 surgical units, with bulk data submitted via a web-based collection system established by the NCD [1]. General thoracic surgery departments reported 91,626 procedures in 2019 (Table 7), which is 2.2 times more than that in 2000 and approximately 14,500 more procedures than that in 2014 (Fig. 2).

**Table 7:** Total cases of general thoracic surgery during 2019

	Cases	%
Benign pulmonary tumor	2543	2.8
Primary lung cancer	48,052	52.4
Other primary malignant pulmonary tumor	432	0.5
Metastatic pulmonary tumor	9329	10.2
Tracheal tumor	117	0.1
Mesothelioma	682	0.7
Chest wall tumor	689	0.8
Mediastinal tumor	5861	6.4
Thymectomy for MG without thymoma	162	0.2
Inflammatory pulmonary disease	2,358	2.6
Empyema	3298	3.6
Bullous disease excluding pneumothorax	394	0.4
Pneumothorax	15,082	16.5
Chest wall deformity	208	0.2
Diaphragmatic hernia including traumatic	36	0.0
Chest trauma excluding diaphragmatic hernia	469	0.5
Lung transplantation	92	0.1
Others	1822	2.0
Total	91,626	100.0

In 2019, 48,052 procedures for primary lung cancer had been performed which continued to increase annually. Accordingly, the number of procedures in 2019 was 2.6 times higher than that in 2000, with lung cancer procedures accounting for 52% of all general thoracic surgeries.

Information about the number of video-assisted thoracoscopic surgery (VATS), which is defined as surgical procedures using a skin incision less than 8 cm including a mini-thoracotomy (hybrid) approach, have been available since the 2015 annual report. Tables 8, 9, 11, 14, 15, 16, 18, 19, 20, 21, 22, and 24, 25, 26 present the number of VATS procedures for benign pulmonary tumors, primary lung cancer, metastatic pulmonary tumor, chest wall tumor, mediastinal tumor, thymectomy for myasthenia gravis, non-neoplastic disease, empyema, descending necrotizing mediastinitis, bullous diseases, diaphragmatic hernia, chest trauma and the total number of VATS procedures in 2019, respectively.

**Table 8:** Benign pulmonary tumor

	Cases	30-Day mortality		Hospital mortality	By VATS
		Hospital	After discharge		
Benign pulmonary tumor					
Hamartoma	565				549
Sclerosing hemangioma	108				102
Papilloma	27				27
Mucous gland adenoma bronchial	10				10
Fibroma	136				132
Lipoma	7				6
Neurogenic tumor	11				10
Clear cell tumor	2				2
Leiomyoma	24				23
Chondroma	3				1
Inflammatory myofibroblastic tumor	0				0
Pseudolymphoma	18				17
Histiocytosis	17				16
Teratoma	6				6
Others	1609	1 (0.1)		3 (0.2)	1536
Total	2543	1 (0.04)		3 (0.12)	2437

( ), Mortality %

**Table 9:** Primary malignant pulmonary tumor

	Cases	30-Day mortality		Hospital mortality	VATS
		Hospital	After discharge		
2. Primary malignant pulmonary tumor	48,484	109 (0.2)	66 (0.1)	240 (0.5)	38,158
Lung cancer	48,052	107 (0.2)	66 (0.1)	238 (0.5)	38,158
Adenocarcinoma	34,290	49 (0.1)	39 (0.11)	99 (0.3)	
Squamous cell carcinoma	8,642	47 (0.5)	23 (0.3)	107 (1.2)	
Large cell carcinoma	311				
LCNEC	611	1 (0.2)	1 (0.2)	5 (0.8)	
Small cell carcinoma	776	1 (0.1)	3 (0.4)	5 (0.6)	
Adenosquamous carcinoma	538	1 (0.2)		3 (0.6)	
Carcinoma with pleomorphic, sarcomatoid or sarcomatous elements	540	4 (0.7)		6 (1.1)	
Carcinoid	282			1 (0.4)	
Carcinomas of salivary-gland type	45				
Unclassified	42	1 (2.4)		2 (4.8)	
Multiple lung cancer	1,623	3 (0.2)		7 (0.4)	
Others	352			3 (0.9)	
Unknown		1	1	2	
Wedge resection	8,532	10 (0.1)	9 (0.1)	28 (0.3)	7,770
Segmental excision	5,467	5 (0.1)	6 (0.11)	19 (0.3)	4,674
(*Sleeve segmental excision*)	20				12
Lobectomy	33,445	85 (0.3)	49 (0.15)	177 (0.5)	25,487
(*Sleeve lobectomy*)	483	8 (1.7)	3 (0.6)	9 (1.9)	70
Pneumonectomy	278	4 (1.4)		10 (3.6)	31
(*Sleeve pneumonectomy*)	5				0
Other bronchoplasty	38			1 (2.6)	5
Pleuropneumonectomy	1				0
Others	291	4 (1.4)	3 (1.0)	5 (1.7)	191
Unknown	0				
Sarcoma	47	2 (4.3)		2 (4.3)	
AAH	120				
Others	265				

( ), Mortality %

A total of 2543 procedures for benign pulmonary tumors had been conducted in 2019 (Table 8). Hamartomas were the most frequent benign pulmonary tumors diagnosed, with 2437 patients (96%) undergoing VATS.

Tables 9 and 10 show additional information on primary malignant pulmonary tumors. Accordingly, the most frequently diagnosed lung cancer subtype was adenocarcinoma (71% of all lung cancers), followed by squamous cell carcinoma (18%). Sublobar resection was performed in 13,999 lung cancer cases (29% of all cases) and lobectomy in 33,455 cases (70% of all cases). Sleeve lobectomy was performed in 483 cases, while pneumonectomy was required in 278 cases (0.6% of all cases). VATS lobectomy was performed in 25,487 cases of lung cancer (76% of all lobectomy cases). Patients aged ≥ 80 years who underwent lung cancer surgery accounted for 6739 (14%). Among those who died within 30 days postoperatively, 107 and 66 died before and after hospital discharge, respectively. Overall, 173 patients died within 30 days postoperatively (30-day mortality rate, 0.4%), while 238 died before discharge (hospital mortality rate, 0.5%). Moreover, 30-day mortality rates according to the procedure were 0.1%, 0.3%, and 1.4% for segmentectomy, lobectomy, and pneumonectomy, respectively. Interstitial pneumonia had been the leading cause of death after lung cancer surgery, followed by pneumonia, respiratory failure, and cardiovascular events.

**Table 10:** Details of lung cancer operations

TNM	
c-Stage	Cases
IA1	8727
IA2	13,908
IA3	8400
IB	5295
IIA	1687
IIB	4018
IIIA	2694
IIIB	457
IIIC	15
IVA	383
IVB	80
NA	2,388
Total	48,052

Sex	Cases
Male	29,065
Female	18,987
Total	48,052

Cause of death	Cases
Cardiovascular	30
Pneumonia	50
Pyothorax	2
Bronchopleural fistula	14
Respiratory failure	32
Pulmonary embolism	2
Interstitial pneumonia	87
Brain infarction or bleeding	10
Others	70
Unknown	10
Total	307

p-Stage	Cases
0 (pCR)	3532
IA1	9737
IA2	10,819
IA3	5323
IB	6496
IIA	1343
IIB	4712
IIIA	3949
IIIB	770
IIIC	10
IVA	942
IVB	92
NA	327
Total	48,052

Age (y)	Cases
<20	19
20–29	48
30–39	259
40–49	1299
50–59	3987
60–69	12,825
70–79	22,874
80–89	6,614
≥90	125
NA	2
Total	48,052

Table 11 shows the procedures for metastatic pulmonary tumors, of which 9329 were performed in 2019. Among such procedures, the most frequent primary tumor was colorectal cancer (51% of all cases).

**Table 11:** Metastatic pulmonary tumor

	Cases	30-Day mortality		Hospital mortality	VATS
		Hospital	After discharge		
3. Metastatic pulmonary tumor	9329	9 (0.1)	6 (0.06)	16 (0.2)	8709
Colorectal	4379	3 (0.07)		5 (0.1)	4083
Hepatobiliary/Pancreatic	525	1 (0.2)		1 (0.2)	497
Uterine	516	2 (0.4)		2 (0.4)	490
Mammary	568				547
Ovarian	75				72
Testicular	57				53
Renal	770				732
Skeletal	144				133
Soft tissue	246		3 (1.2)		229
Otorhinolaryngological	559				525
Pulmonary	449	1 (0.2)	1 (0.2)	2 (0.4)	386
Others	1041	2 (0.2)	2 (0.2)	6 (0.6)	962

( ), Mortality %

A total of 117 procedures for tracheal tumors, including 60, 30, and 27 cases of primary malignant, metastatic, and benign tracheal tumors, respectively, were performed in 2019. Further, 35 patients underwent sleeve resection and reconstruction (Table 12).

**Table 12:** Tracheal tumor

	Cases	30-Day mortality		Hospital mortality
		Hospital	After discharge	
4. Tracheal tumor	117	2 (1.7)	3 (2.6)	5 (4.3)
A. Primary malignant tumor				
Histological classification				
Squamous cell carcinoma	14			2 (14.3)
Adenoid cystic carcinoma	31		1 (3.2)	
Mucoepidermoid carcinoma	1			
Others	14			
Total	60		1 (1.7)	2 (3.3)
B. Metastatic/invasive malignant tumor, e.g. invasion of thyroid cancer				
	30	2 (6.7)	2 (6.7)	3 (10.0)
C. Benign tracheal tumor				
Histological classification				
Papilloma	3			
Adenoma	2			
Neurofibroma	1			
Chondroma	1			
Leiomyoma	2			
Others	18			
Histology unknown	0			
Total	27	0	0	0
Operation				
Sleeve resection with reconstruction	35		1 (2.9)	1 (2.9)
Wedge with simple closure	4			
Wedge with patch closure	0			
Total laryngectomy with tracheostomy	0			
Others	1			
Unknown	0			
Total	40	0	1 (2.5)	1 (2.5)

( ), Mortality %

Overall, 682 pleural tumors had been diagnosed in 2019 (Table 13), with diffuse malignant pleural mesothelioma as the most frequent histologic diagnosis. Total pleurectomy was performed in 140 cases and extrapleural pneumonectomy in 43 cases. The 30-day mortality rate was 0% and 2.3% after total pleurectomy and extrapleural pneumonectomy, respectively, both of which had better outcomes than previously reported.

**Table 13:** Tumor of pleural origin

Histological classification	Cases	30-Day mortality		Hospital mortality
		Hospital	After discharge	
5. Tumor of pleural origin				
Solitary fibrous tumor	133			
Diffuse malignant pleural mesothelioma	292	2 (0.7)		10 (3.4)
Localized malignant pleural mesothelioma	37			1 (2.7)
Others	220	2 (0.9)		4 (1.8)
Total	682	4 (0.6)		15 (2.2)

Operative procedure	Cases	30-Day mortality		Hospital mortality
		Hospital	After discharge	
Extrapleural pneumonectomy	43	1 (2.3)		3 (7.0)
Total pleurectomy	140			3 (2.1)
Others	109	1 (0.9)		4 (3.7)
Total	292	2 (0.7)		10 (3.4)

( ), Mortality %

Overall, 689 chest wall tumor resections had been performed in 2019, including 116, 209, and 364 cases of primary malignant, metastatic, and benign tumors, respectively (Table 14).

**Table 14:** Chest wall tumor

	Cases	30-Day mortality		Hospital mortality	VATS
		Hospital	After discharge		
6. Chest wall tumors					
Primary malignant tumor	116	1 (0.9)	1 (0.9)	1 (0.9)	56
Metastatic malignant tumor	209				84
Benign tumor	364				283
Total	689	1 (0.1)	1 (0.1)	1 (0.1)	423

( ), Mortality %

In 2019, 5,881 mediastinal tumors were resected, which is 10% higher compared to that in the previous year (Table 15). Thymic epithelial tumors, including 2280 thymomas, 351 thymic carcinomas, and 44 thymic carcinoids, were the most frequently diagnosed mediastinal tumor subtype in 2019.

**Table 15:** Mediastinal tumor

	Cases	30-Day mortality		Hospital mortality	By VATS
		Hospital	After discharge		
7. Mediastinal tumor	5881	2 (0.03)	10 (0.17)	10 (0.2)	4599
Thymoma*	2280		2 (0.1)	2 (0.1)	1612
Thymic cancer	351		1 (0.3)	1 (0.3)	222
Thymus carcinoid	44				25
Germ cell tumor	111				66
*Benign*	89				59
*Malignant*	22				7
Neurogenic tumor	526	1 (0.2)			490
Congenital cyst	1376		1 (0.1)	1 (0.1)	1293
Goiter	96			0	36
Lymphatic tumor	160			0	125
Excision of pleural recurrence of thymoma	30			0	23
Thymolipoma	15	1 (6.7)		0	9
Others	892		6 (0.7)	6 (0.7)	698

( ), Mortality %

A total of 499 patients underwent thymectomy for myasthenia gravis (Table 16), among which 337 procedures were associated with thymoma.

**Table 16:** Thymectomy for myasthenia gravis

	Cases	30-Day mortality		Hospital mortality	By VATS
		Hospital	After discharge		
8. Thymectomy for myasthenia gravis	499	1 (0.2)	0	3 (0.6)	298
With thymoma	337	0	0	0	202

( ), Mortality %

Overall, 23,717 patients underwent procedures for non-neoplastic disease. Accordingly, 2358 patients underwent lung resection for inflammatory lung diseases (Tables 17, 18), among which 475 and 336 patients were associated with mycobacterial and fungal infections, respectively. Procedures for inflammatory nodules were performed in cases where lung cancer was suspected preoperatively (928 cases, 39%).

**Table 17:** Operations for non-neoplastic diseases:A+B+C+D+E+F+G+H+I

	Cases	30-Day mortality		Hospital mortality
		Hospital	After discharge	
9. Operations for non-neoplastic diseases	23,717	221 (0.9)	33 (0.1)	491 (2.1)

**Table 18:** A. Inflammatory pulmonary disease

	Cases	30-Day mortality		Hospital mortality	VATS
		Hospital	After discharge		
A. Inflammatory pulmonary disease	2358	7 (0.3)	1 (0.0)	15 (0.6)	2130
Tuberculous infection	41				35
Mycobacterial infection	475	1 (0.2)		2 (0.4)	425
Fungal infection	336	1 (0.3)		2 (0.6)	267
Bronchiectasis	52				42
Tuberculous nodule	70			1 (1.4)	69
Inflammatory pseudotumor	928	2 (0.2)	1 (0.1)	4 (0.4)	876
Interpulmonary lymph node	66				65
Others	390	3 (0.8)		6 (1.5)	351

( ), Mortality %

A total of 3298 procedures were performed for empyema (Table 19), among which 2597 (77%) were acute and 701 were chronic. Further, bronchopleural fistulas developed in 478 and 320 patients with acute and chronic empyema, respectively. The hospital mortality rate was 13% among patients with acute empyema with fistula.

**Table 19:** B. Empyema

	Cases	30-Day mortality			Hospital mortality	By VATS
		Hospital	After discharge			
Acute empyema	2597	53 (2.0)	3 (0.1)		144 (5.5)	2,233
With fistula	478	10 (2.1)			62 (13.0)	287
Without fistula	2096	43 (2.1)	3 (0.1)		81 (3.9)	1,925
Unknown	23				1 (4.3)	21
Chronic empyema	701	20 (2.9)	2 (0.3)		55 (7.8)	404
With fistula	320	16 (5.0)	1 (0.3)		36 (11.3)	127
Without fistula	342	3 (0.9)	1 (0.3)		18 (5.3)	246
Unknown	39	1 (2.6)			1 (2.6)	31
Total	3298	73 (2.2)	5 (0.2)		199 (6.0)	2637

( ), Mortality %

Further, 93 operations were performed for descending necrotizing mediastinitis (Table 20), with a hospital mortality rate of 4.3%.

**Table 20:** C. Descending necrotizing mediastinitis

	Cases	30-Day mortality		Hospital mortality	VATS
		Hospital	After discharge		
C. Descending necrotizing mediastinitis	93	2 (2.2)		4 (4.3)	78

( ), Mortality %

A total of 394 procedures were conducted for bullous diseases (Table 21), while only 13 patients underwent lung volume reduction surgery.

**Table 21:** D. Bullous diseases

	Cases	30-Day mortality		Hospital mortality	VATS
		Hospital	After discharge		
D. Bullous diseases	394	1 (0.3)		1 (0.3)	359
Emphysematous bulla	296	1 (0.3)		1 (0.3)	270
Bronchogenic cyst	22				20
Emphysema with LVRS	13				13
Others	63				56

( ), Mortality %

*LVRS* lung volume reduction surgery

A total of 15,082 procedures were performed for pneumothorax (Table 22). Among the 11,200 procedures for spontaneous pneumothorax, 2762 (25%) were bullectomies alone, while 7714 (69%) required additional procedures, such as coverage with artificial material, as well as parietal pleurectomy. A total of 3,882 procedures for secondary pneumothorax were performed, with chronic obstructive pulmonary disease (COPD) being the most prevalent associated disease (2693 cases, 69%). The hospital mortality rate for secondary pneumothorax associated with COPD was 1.7%.

**Table 22:** E. Pneumothorax

Cases	30-Day mortality		Hospital mortality	VATS
	Hospital	After discharge		
15,082	78 (0.5)	22 (0.1)	163 (1.1)	14,711

Spontaneous pneumothorax					
Operative procedure	Cases	30-Day mortality		Hospital mortality	VATS
		Hospital	After discharge		
Bullectomy	2762	5 (0.2)	1 (0.0)	6 (0.2)	2702
Bullectomy with additional procedure	7714	4 (0.1)	1 (0.01)	11 (0.1)	7596
Coverage with artificial material	7442	4 (0.1)	1 (0.01)	11 (0.1)	7327
Parietal pleurectomy	33				33
Coverage and parietal pleurectomy	71				71
Others	168				165
Others	721	2 (0.3)		10 (1.4)	688
Unknown	3				3
Total	11,200	11 (0.1)	2 (0.0)	27 (0.2)	10,989

Secondary pneumothorax					
Associated disease	Cases	30-Day mortality		Hospital mortality	VATS
		Hospital	After discharge		
COPD	2693	25 (0.9)	9 (0.3)	57 (2.1)	2,611
Tumorous disease	168	11 (6.5)	4 (2.4)	19 (11.3)	158
Catamenial	164				162
LAM	49	0			48
Others (excluding pneumothorax by trauma)	808	31 (3.8)	7 (0.9)	60 (7.4)	745

Operative procedure	Cases	30 Day mortality		Hospital mortality	VATS
		Hospital	After discharge		
Bullectomy	627	7 (1.1)	3 (0.5)	13 (2.1)	615
Bullectomy with additional procedure	2285	21 (0.9)	8 (0.4)	42 (1.8)	2233
Coverage with artificial material	2190	19 (0.9)	8 (0.4)	39 (1.8)	2140
Parietal pleurectomy	6				6
Coverage and parietal pleurectomy	24	1 (4.2)		1 (4.2)	22
Others	65	1 (1.5)		2 (3.1)	65
Others	966	39 (4.0)	9 (0.9)	81 (8.4)	872
Unknown	4			0	4
Total	3882	67 (1.7)	20 (0.5)	136 (3.5)	3724

( ), Mortality %

The 2019 survey reported 208 procedures for chest wall deformity (Table 23). However, this may have been underestimated because the Nuss procedure for pectus excavatum was more likely performed in pediatric surgery centers not associated with the Japanese Association for Thoracic Surgery.

**Table 23:** F. Chest wall deformity

	Cases	30-Day mortality				Hospital mortality	
		Hospital		After discharge			
F. Chest wall deformity	208						
Funnel chest	196						
Others	12						

( ), Mortality %

Surgical treatment for diaphragmatic hernia was performed in 36 patients (Table 24). This figure may have been underestimated because procedures may have been classified as gastrointestinal surgery.

**Table 24:** G. Diaphragmatic hernia

	Cases	30-Day mortality		Hospital mortality	VATS
		Hospital	After discharge		
G. Diaphragmatic hernia	36				21
Congenital	6				5
Traumatic	10				4
Others	20				12

( ), Mortality %

The survey reported 469 procedures for chest trauma, excluding iatrogenic injuries (Table 25), with a hospital mortality rate of 5.5%.

**Table 25:** H. Chest trauma

	Cases	30-Day mortality		Hospital mortality	VATS
		Hospital	After discharge		
H. Chest trauma	469	26 (5.5)	1 (0.2)	29 (6.2)	302

( ), Mortality %

Table 26 summarizes the procedures for other diseases, including 110 and 118 cases of arteriovenous malformation and pulmonary sequestration, respectively.

**Table 26:** I. Other respiratory surgery

	Cases	30-Day mortality		Hospital mortality	VATS
		Hospital	After discharge		
I. Other respiratory surgery	1783	34 (1.9)	4 (0.2)	80 (4.5)	1400
Arteriovenous malformation*	110		1 (0.9)	1 (0.9)	104
Pulmonary sequestration	118				105
Postoperative bleeding ・air leakage	555	9 (1.6)		34 (6.1)	404
Chylothorax	85	2 (2.4)		2 (2.4)	77
Others	915	23 (2.5)	3 (0.3)	43 (4.7)	710

( ), Mortality %

A total of 92 lung transplantations were performed in 2019 (Table 27), among which 80 and 12 were from brain-dead and living-related donors, respectively.

**Table 27:** Lung transplantation

10. Lung transplantation	Cases	30-Day mortality		Hospital mortality
		Hospital	After discharge	
Lung transplantation from brain-dead donor	44			2 (4.5)
Bilateral lung transplantation from brain-dead donor	36	1 (2.8)		1 (2.8)
Lung transplantation from living donor	12			1 (8.3)
Total lung transplantation	92	1 (1.1)		4 (4.3)
Donor of living donor lung transplantation	23			

( ), Mortality %

The number of VATS procedures has continued to increase annually, ultimately reaching 77,059 (84% of all general thoracic surgeries) in 2019 (Table 28).

**Table 28:** Video-assisted thoracic surgery

	Cases	30-Day mortality		Hospital mortality
		Hospital	After discharge	
11. Video-assisted thoracic surgery	77,059	221 (0.3)	78 (0.1)	455 (0.6)

( ), Mortality % (including thoracic sympathectomy 160)

Tables 29, 30, 31, 32 present the details regarding tracheobronchoplasty, pediatric surgery, and combined resection of neighboring organs.

**Table 29:** Tracheobronchoplasty

	Cases	30-Day mortality		Hospital mortality
		Hospital	After discharge	
12. Tracheobronchoplasty	787	13 (1.7)	6 (0.8)	21 (2.7)
Trachea	52	1 (1.9)	1 (1.9)	2 (3.8)
Sleeve resection with reconstruction	37	0	1 (2.7)	1 (2.7)
Wedge with simple closure	6	0	0	0
Wedge with patch closure	0	0	0	0
Total laryngectomy with tracheostomy	0	0	0	0
Others	9	1 (11.1)	0	1 (11.1)
Carinal reconstruction	23	2 (8.7)	0	2 (8.7)
Sleeve pneumonectomy	5	0	0	0
Sleeve lobectomy	486	6 (1.2)	3 (0.6)	7 (1.4)
Sleeve segmental excision	25	0	0	0
Bronchoplasty without lung resection	22	0	0	1 (4.5)
Others	174	4 (2.3)	2 (1.1)	9 (5.2)

( ), Mortality %

**Table 30:** Pediatric surgery

	Cases	30-Day mortality		Hospital mortality
		Hospital	After discharge	
13. Pediatric surgery	341	7 (2.1)		9 (2.6)

( ), Mortality %

**Table 31:** Combined resection of neighboring organ(s)

	Cases	30-Day mortality		Hospital mortality
		Hospital	After discharge	
14. Combined resection of neighboring organ(s)	1355	3 (0.2)		15 (1.1)

Organ resected	Cases	30-Day mortality		Hospital mortality
		Hospital	After discharge	
A. Primary lung cancer				
Aorta	10			
Superior vena cava	22	1 (4.5)		2 (9.1)
Brachiocephalic vein	4			
Pericardium	82	1 (1.2)		2 (2.4)
Pulmonary artery	124	3 (2.4)		4 (3.2)
Left atrium	19			
Diaphragm	65			1 (1.5)
Chest wall (including ribs)	327			5 (1.5)
Vertebra	12			
Esophagus	3			
Total	668	5 (0.7)	0	14 (2.1)
B. Mediastinal tumor				
Aorta	2	0	0	2 (100.0)
Superior vena cava	56	0	0	1 (1.8)
Brachiocephalic vein	121	0	0	1 (0.8)
Pericardium	345	0	0	2 (0.6)
Pulmonary artery	6	0	0	1 (16.7)
Left atrium	1	0	0	0
Diaphragm	39	0	0	0
Chest wall (including ribs)	10	0	0	0
Vertebra	11	0	0	0
Esophagus	4	0	0	0
Lung	510	0	0	2 (0.4)
Total	1105	0	0	9 (0.8)

( ), Mortality %

**Table 32:** Operation of lung cancer invading the chest wall of the apex

	Cases	30-Day mortality		Hospital mortality
		Hospital	After discharge	
15. Operation of lung cancer invading the chest wall of the apex	782	3 (0.4)	1 (0.1)	11 (1.4)

( ), Mortality %

Includes tumors invading the anterior apical chest wall and posterior apical chest wall (superior sulcus tumor, so called Pancoast type)

### (C) Esophageal surgery

In 2018, the data collection method for esophageal surgery had been modified from self-reports using questionnaire sheets following each institution belonging to the Japanese Association for Thoracic Surgery to an automatic package downloaded from the NCD in Japan. Consequently, the registry excluded data for non-surgical cases with esophageal diseases. Furthermore, data regarding the histological classification of malignant tumors, multiple primary cancers, and mortality rates for cases with combined resection of other organs could not be registered because they were not included in the NCD. Instead, detailed data regarding postoperative surgical and non-surgical complications were collected from the NCD. Moreover, data regarding surgeries for corrosive esophageal strictures and salvage surgeries for esophageal cancer had been exceptionally registered by participating institutions.

**Fig. 3:**
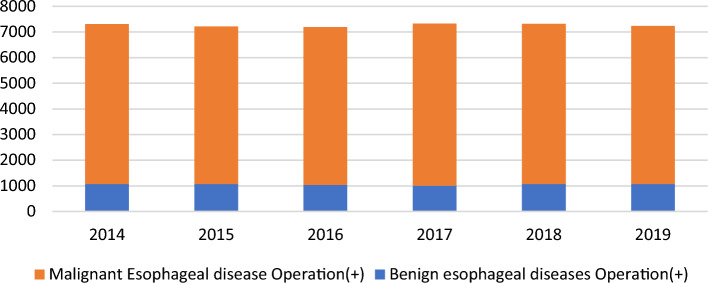
Annual trend of in-patients with esophageal diseases

Throughout 2019, 7235 patients underwent surgery for esophageal diseases (1074 and 6161 for benign and malignant esophageal diseases, respectively) from 499 institutions across Japan. Among them, 296 (59.3%) and 379 (76.0%) institutions performed surgeries for benign and malignant esophageal diseases, respectively. Among 379 institutions performing surgeries for malignant esophageal diseases, 53 (14.0%) had ≥ 30 patients, while 299 (78.9%) had < 20 patients (i.e., 1–19 patients) who underwent esophageal surgeries within 2019 (Table 33). This distribution was different from that in 2018 (10.4% and 87.3%, respectively), suggesting that hospital centralization for esophagectomy might be gradually proceeding in Japan. Annual trends among registered in-patients with benign or malignant esophageal diseases have remained unchanged for the past 6 years (Fig. 3).

**Table 33:** Distribution of number of esophageal operations in 2019 in each institution

Esophageal surgery			
Number of operations in 2019	Benign esophageal diseases	Malignant Esophageal disease	Benign+Malignant
0	203	120	85
1–4	240	139	145
5–9	42	77	82
10–19	9	83	84
20–29	1	27	44
30–39	0	18	14
40–49	2	9	13
≧ 50	2	26	32
Total	499	499	499

Concerning benign esophageal diseases (Table 34), thoracoscopic and/or laparoscopic surgeries were performed in 91.1% (72/79), 84.8% (451/532), 46.8% (22/47), and 44.6% (90/202) of patients with esophagitis (including esophageal ulcer), hiatal hernia, benign tumors, and achalasia, respectively. Conversely, 95.7% (134/140) of patients with spontaneous rupture of the esophagus underwent open surgery. Hospital mortality rates within 30 postoperative days were 0.8% (4/532), 5.7% (8/140), 16.7% (1/6), 1.3% (1/79), and 3.3% (1/30) for hiatal hernia, esophagus, esophagi-tracheal fistula, esophagitis, including esophageal ulcer, and corrosive stricture of the esophagus, respectively.

**Table 34:** Benign esophageal diseases

	Operation (+)				T/L*3			
	Cases	Hospital mortality			Cases	Hospital mortality		
		~30 days	31–90 days	Total (including after 91 days mortality)		~30 days	31–90 days	Total (including after 91 days mortality)
1. Achalasia	202				90			
2. Benign tumor	47				22			
3. Diverticulum	38				6			
4. Hiatal hernia	532	4 (0.8)	1 (0.2)	5 (0.9)	451	2 (0.4)		2 (0.4)
5. Spontaneous rupture of the esophagus	140	8 (5.7)	3 (2.1)	11 (7.9)	6			
6. Esophago-tracheal fistula	6	1 (16.7)		1 (16.7)	1	1 (100.0)		1 (100.0)
7. Esophagitis, Esophageal ulcer	79	1 (1.3)	1 (1.3)	2 (2.5)	72	1 (1.4)	1 (1.4)	2 (2.8)
8. Corrosive stricture of the esophagus	30	1 (3.3)	1 (3.3)	2 (6.7)	18			
Total	1074	15 (1.4)	6 (0.6)	21 (2.0)	666	4 (0.6)	1 (0.2)	5 (0.8)

( ), Mortality %

*T/L* Thoracoscopic and/or laparoscopic

The most common tumor location for malignant esophageal diseases was the thoracic esophagus (Table 35). Among 6161 cases with esophageal malignancies, esophagectomy for superficial and advanced cancers was performed in 2400 (39.0%) and 3761 (61.0%), respectively. Hospital mortality rates within 30 days after esophagectomy were 0.3% and 1.1% for patients with superficial and advanced cancer, respectively.

**Table 35:** Malignant esophageal disease

	Operation (+)				Thoracoscopic and/or laparscopic procedure				
	Cases	Hospital mortality			Cases	Conversion to thoracotomy	Hospital mortality		
		~30 days	31–90 days	Total (including after 91 days mortality)			~30 days	31–90 days	Total (including after 91 days mortality)
Location									
(1) Cervical esophagus	155		3 (1.9)	3 (1.9)	57				
(2) Thoracic esophagus	5142	38 (0.7)	36 (0.7)	74 (1.4)	4145	39 (0.9)	28 (0.7)	26 (0.6)	54 (1.3)
(3) Abdominal esophagus	508	6 (1.2)	4 (0.8)	10 (2.0)	350	5 (1.4)		1 (0.3)	6 (1.7)
Total	5805	44 (0.8)	43 (0.7)	87 (1.5)	4552	39 (0.9)	33 (0.7)	27 (0.6)	60 (1.3)
Tumor depth									
(A) Superficial cancer (T1)									
(1) Transhiatal esophagectomy	13				0				
(2) Mediastinoscopic esophagectomy and reconstruction	126				126				
(3) Transthoracic (rt.) esophagectomy and reconstruction	**1659**	5 (0.3)	5 (0.3)	10 (0.6)	**1409**	6 (0.4)	4 (0.3)	5 (0.4)	9(0.6)
(4) Transthoracic (lt.) esophagectomy and reconstruction	29				14				
(5) Cervical esophageal resection and reconstruction	26				0				
(6) Robot-assisted esophagectomy and reconstruction	295	2 (0.7)		2 (0.7)	294	1 (0.3)	2 (0.7)		2 (0.7)
(7) Others	19				0				
(8) Esophagectomy without reconstruction	233				0				
Subtotal	**2400**	7 (0.3)	5 (0.2)	12 (0.5)	**1843**	7 (0.4)	6 (0.3)	5 (0.3)	11 (0.6)
(B) Advanced cancer (T2-T4)									
(1) Transhiatal esophagectomy	21	1 (4.8)	2 (9.5)	3 (14.3)	0				
(2) Mediastinoscopic esophagectomy and reconstructio	112	1 (0.9)	2 (1.8)	3 (2.7)	112		1 (0.9)	2 (1.8)	3 (2.7)
(3) Transthoracic (rt.) esophagectomy and reconstruction	**2910**	30 (1.0)	26 (0.9)	56 (1.9)	**2178**	27 (1.2)	22 (1.0)	15 (0.7)	37 (1.7)
(4) Transthoracic (lt.) esophagectomy and reconstruction	74	1 (1.4)	1 (1.4)	2 (2.7)	23		1 (4.3)		1 (4.3)
(5) Cervical esophageal resection and reconstruction	68				0				
(6) Robot-assisted esophagectomy and reconstruction	374	2 (0.5)	4 (1.1)	6 (1.6)	374	4 (1.1)	2 (0.5)	4 (1.1)	6 (1.6)
(7) Others	59	1 (1.7)	1 (1.7)	2 (3.4)	0				
(8) Esophagectomy without reconstruction	143	4 (2.8)	6 (4.2)	10 (7.0)	0				
Subtotal	**3761**	40 (1.1)	42 (1.1)	82 (2.2)	**2687**	31 (1.2)	26 (1.0)	21 (0.8)	47 (1.7)
Total	**6161**	47 (0.8)	47 (0.8)	94 (1.5)	**4530**	38 (0.8)	32 (0.7)	26(0.6)	58 (1.3)

	Cases	Overall morbidity	Morbidity ≥CD III	Surgical complications					
				Surgical site infection			Anastomotic leakage	Recurrent nerve palsy	Wound dehiscence
				Superficial incision	Deep incision	Organ space			
Location									
(1) Cervical esophagus	155	104 (67.1)	56 (36.1)	15 (9.7)	8 (5.2)	10 (6.5)	24 (15.5)	20 (12.9)	2 (1.3)
(2) Thoracic esophagus	5142	2950 (57.4)	1148 (22.3)	391 (7.6)	205 (4.0)	459 (8.9)	707 (13.7)	774 (15.1)	85 (1.7)
(3) Abdominal esophagus	508	253 (49.8)	97(19.1)	27 (5.3)	10 (2.0)	46 (9.1)	75 (14.8)	40 (7.9)	5 (1.0)
Total	5805	3307 (57.0)	1301 (22.4)	433 (7.5)	223 (3.8)	515 (8.9)	806 (13.9)	834 (14.4)	92 (1.6)
Tumor depth									
(A) Superficial cancer (T1)									
(1) Transhiatal esophagectomy	13	9 (69.2)	7 (53.8)	3 (23.1)	2 (15.4)	2 (15.4)	2 (15.4)	1 (7.7)	0
(2) Mediastinoscopic esophagectomy and reconstructio	126	77 (61.1)	28 (22.2)	9 (7.1)	3 (2.4)	7 (5.6)	19 (15.1)	35 (27.8)	2 (1.6)
(3) Transthoracic (rt.) esophagectomy and reconstruction	1659	891 (53.7)	325 (19.6)	116 (7.0)	58 (3.5)	146 (8.8)	236 (14.2)	222 (13.4)	25 (1.5)
(4) Transthoracic (lt.) esophagectomy and reconstruction	29	15 (51.7)	6 (20.7)	2 (6.9)	2 (6.9)	5 (17.2)	4 (13.8)	4 (13.8)	2 (6.9)
(5) Cervical esophageal resection and reconstruction	26	19 (73.1)	10 (38.5)	1 (3.8)	2 (7.7)	1 (3.8)	2 (7.7)	6 (23.1)	1 (3.8)
(6) Robot-assisted esophagectomy and reconstruction	295	172 (58.3)	75 (25.4)	21 (7.1)	15 (5.1)	32 (10.8)	47 (15.9)	46 (15.6)	3 (1.0)
(7) Others	19	10 (52.6)	5 (26.3)	1 (5.3)	4 (21.1)	6 (31.6)	1(5.3)	0	
(8) Esophagectomy without reconstruction	233	34 (14.6)	9 (3.9)						
Subtotal	**2400**	1227 (51.1)	465 (19.4)	153 (6.4)	82 (3.4)	197 (8.2)	316 (13.2)	315 (13.1)	33 (1.4)
(B) Advanced cancer (T2-T4)									
(1) Transhiatal esophagectomy	21	12 (57.1)	9 (42.9)	7 (33.3)	5 (23.8)	2 (9.5)	3 (14.3)	1 (4.8)	2 (9.5)
(2) Mediastinoscopic esophagectomy and reconstructio	112	74 (66.1)	27 (24.1)	11 (9.8)	4 (3.6)	8 (7.1)	27 (24.1)	24 (21.4)	2 (1.8)
(3) Transthoracic (rt.) esophagectomy and reconstruction	2910	1693 (58.2)	675 (23.2)	222 (7.6)	120 (4.1)	275 (9.5)	404 (13.9)	427 (14.7)	45 (1.5)
(4) Transthoracic (lt.) esophagectomy and reconstruction	74	40 (54.1)	18 (24.3)	6 (8.1)	3 (4.1)	3 (4.1)	7 (9.5)	4 (5.4)	3 (4.1)
(5) Cervical esophageal resection and reconstruction	68	43 (63.2)	21 (30.9)	9 (13.2)	3 (4.4)	3 (4.4)	8 (11.8)	8 (11.8)	1 (1.5)
(6) Robot-assisted esophagectomy and reconstruction	374	218 (58.3)	82 (21.9)	22 (5.9)	5 (1.3)	21 (5.6)	35 (9.4)	52 (13.9)	5 (1.3)
(7) Others	59	20 (33.9)	7 (11.9)	1 (1.7)		4 (6.8)	5 (8.5)		
(8) Esophagectomy without reconstruction	143	84 (58.7)	42 (29.4)						
Subtotal	**3761**	2184 (58.1)	881 (23.4)	278 (7.4)	140 (3.7)	316 (8.4)	489 (13.0)	516 (13.7)	58 (1.5)
Total	6161	3411 (55.4)	1346 (21.8)	431 (7.0)	222 (3.6)	513 (8.3)	805 (13.1)	831 (13.5)	91 (1.5)

	Cases	Nonsurgical complications										
		Pneumonia	Unplanned intubation	Prolonged ventilation>48h	Pulmonary embolism	Atelectasis	Renal failure	CNS events	Cardiac events	Septic shock	Readmission within 30d	Reoperation within 30d
Location												
(1) Cervical esophagus	155	22 (14.2)	10 (6.5)	17 (11.0)	1 (0.6)	6 (3.9)	2 (1.3)	1 (0.6)	1 (0.6)	2 (1.3)	1 (0.6)	23 (14.8)
(2) Thoracic esophagus	5142	815 (15.8)	246 (4.8)	333 (6.5)	42 (0.8)	272 (5.3)	20 (0.4)	29 (0.6)	18 (0.4)	33 (0.6)	125 (2.4)	334 (6.5)
(3) Abdominal esophagus	508	63 (12.4)	19 (3.7)	23 (4.5)	11 (2.2)	25 (4.9)	6 (1.2)	1 (0.2)	3 (0.6)	8 (1.6)	11 (2.2)	32 (6.3)
Total	5805	900 (15.5)	275 (4.7)	373 (6.4)	54 (0.9)	303 (5.2)	28 (0.5)	31 (0.5)	22 (0.4)	43 (0.7)	137 (2.4)	389 (6.7)
Tumor depth												
(A) Superficial cancer (T1)												
(1) Transhiatal esophagectomy	13	2 (15.4)	3 (23.1)	3 (23.1)		1 (7.7)						2 (15.4)
(2) Mediastinoscopic esophagectomy and reconstructio	126	19 (15.1)	5 (4.0)	7 (5.6)		7 (5.6)						4 (3.2)
(3) Transthoracic (rt.) esophagectomy and reconstruction	1659	214 (12.9)	61 (3.7)	84 (5.1)	16 (1.0)	89 (5.4)	7 (0.4)	6 (0.4)	7 (0.4)	7 (0.4)	44 (2.7)	97 (5.8)
(4) Transthoracic (lt.) esophagectomy and reconstruction	29	3 (10.3)	2 (6.9)	2 (6.9)		3 (10.3)		1 (3.4)		1 (3.4)		3 (10.3)
(5) Cervical esophageal resection and reconstruction	26	4 (15.4)	2 (7.7)	3 (11.5)		1 (3.8)	1 (3.8)			1 (3.8)		5 (19.2)
(6) Robot-assisted esophagectomy and reconstruction	295	41 (13.9)	16 (5.4)	17 (5.8)	5 (1.7)	10 (3.4)	1 (0.3)	2 (0.7)		2 (0.7)	4 (1.4)	16 (5.4)
(7) Others	19	1 (5.3)		2 (10.5)		2 (10.5)					1 (5.3)	3 (15.8)
(8) Esophagectomy without reconstruction	233										3 (1.3)	
Subtotal	2400	284 (11.8)	89 (3.7)	118 (4.9)	21 (0.9)	113 (4.7)	9 (0.4)	9 (0.4)	7 (0.3)	11 (0.5)	52 (2.2)	130 (5.4)
(B) Advanced cancer(T2-T4)												
(1) Transhiatal esophagectomy	21	7 (33.3)	3 (14.3)	4 (19.0)		1 (4.8)		1 (4.8)			1 (4.8)	2 (9.5)
(2) Mediastinoscopic esophagectomy and reconstructio	112	21 (18.8)	8 (7.1)	7 (6.3)			5 (4.5)		2 (1.8)	1 (0.9)	1 (0.9)	7 (6.3)
(3) Transthoracic (rt.) esophagectomy and reconstruction	2910	487 (16.7)	146 (5.0)	210 (7.2)	25 (0.9)	155 (5.3)	17 (0.6)	15 (0.5)	11 (0.4)	22 (0.8)	71 (2.4)	200 (6.9)
(4) Transthoracic (lt.) esophagectomy and reconstruction	74	11 (14.9)	3 (4.1)	6 (8.1)		6 (8.1)		1 (1.4)			2 (2.7)	8 (10.8)
(5) Cervical esophageal resection and reconstruction	68	9 (13.2)	2 (2.9)	6 (8.8)		2 (2.9)		1 (1.5)	1 (1.5)	1 (1.5)	1 (1.5)	10 (14.7)
(6) Robot-assisted esophagectomy and reconstruction	374	72 (19.3)	18 (4.8)	18 (4.8)	8 (2.1)	19 (5.1)	2 (0.5)	2 (0.5)	2 (0.5)	6 (1.6)	11 (2.9)	27 (7.2)
(7) Others	59	2 (3.4)	3 (5.1)	2 (3.4)		1 (1.7)			1 (1.7)		1 (1.7)	4 (6.8)
(8) Esophagectomy without reconstruction	143										6 (4.2)	
Subtotal	3761	609 (16.2)	183 (4.9)	253 (6.7)	33 (0.9)	189 (5.0)	19 (0.5)	22 (0.6)	15 (0.4)	30 (0.8)	94 (2.5)	258 (6.9)
Total	6161	893 (14.5)	272 (4.4)	371 (6.0)	54 (0.9)	302 (4.9)	28 (0.5)	31 (0.5)	22 (0.4)	41 (0.7)	146 (2.4)	388 (6.3)

**Fig. 4:**
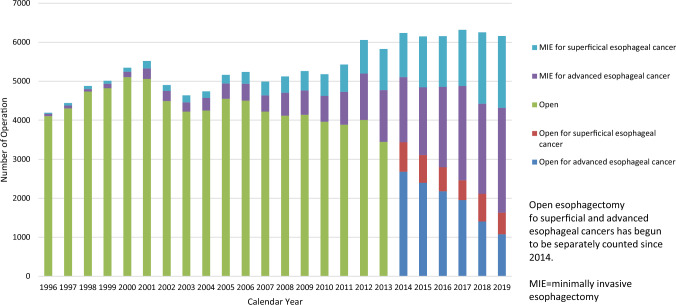
Annual trend of esophagectomy

Among esophagectomy procedures, transthoracic esophagectomy via right thoracotomy or right thoracoscopy was most commonly adopted for patients with superficial (1659/2400, 69.1%) and advanced cancer (2910/3761, 77.4%) (Table 35). Transhiatal esophagectomy, which is commonly performed in Western countries, was adopted in only 13 (0.5%) and 21 (0.6%) patients with superficial and advanced cancer who underwent esophagectomy in Japan, respectively. Thoracoscopic and/or laparoscopic esophagectomy was utilized in 1843 (76.8%) and 2687 (71.4%) patients with superficial and advanced cancer, respectively. Patients who underwent thoracoscopic and/or laparoscopic surgery (minimally invasive esophagectomy: MIE) for superficial or advanced cancer have been increasing, whereas that of open surgery, especially for advanced cancer, has been decreasing annually (Fig. 4). Mediastinoscopic esophagectomy was performed for 126 (5.3%) and 112 (3.0%) patients with superficial and advanced esophageal cancer, respectively. Robot-assisted esophagectomy was performed for 295 (12.3%) and 374 (9.9%) patients with superficial and advanced esophageal cancer, respectively. Patients who underwent robot-assisted surgery are increasing for both superficial and advancer esophageal cancers compared to that in 2018 (6.8% and 4.2% in 2018, respectively). Hospital mortality rates within 30 days after thoracoscopic and/or laparoscopic esophagectomy were 0.3% and 1.0% for patients with superficial and advanced cancer, respectively (Table 35).

Detailed data collection regarding postoperative surgical and non-surgical complications was initiated in 2018. Overall, 1346 (21.8%) of 6161 patients developed grade III or higher complications based on the Clavien–Dindo classification in 2019. Among surgical complications, anastomotic leakage and recurrent nerve palsy occurred in 14.0% and 14.3% of the patients who underwent right transthoracic esophagectomy, in 12.3% and 14.6% of those who underwent robot-assisted esophagectomy, and in 19.7% and 24.8% of those who underwent mediastinoscopic esophagectomy, respectively. Among non-surgical postoperative complications, pneumonia occurred in 14.5% of the patients, 4.4% of whom underwent unplanned intubation. The possible advantage in postoperative pneumonia in patients with mediastinoscopic esophagectomy in 2018 was not observed this year. Postoperative pulmonary embolism occurred in 0.9% of the patients. These complication rates, including the others, were similar to those in 2018.

Salvage surgery following definitive (chemo)radiotherapy was performed in 500 patients, with hospital mortality rates of 0.8% within 30 days postoperatively. Thoracoscopic and/or laparoscopic esophagectomy were performed in 329 (65.8%) patients (47.7% in 2018) (Table 36).

**Table 36:** Salvage surgery

	Operation (+)				Thoracoscopic and/or laparscopic procedure					EMR or ESD
	Cases	Hospital mortality			Cases	Conversion to thoracotomy	Hospital mortality			
		~30 days	31–90 days	Total (including after 91 days mortality)			~30 days	31–90 days	Total (including after 91 days mortality)	
Salvage surgery	500	4 (0.8)	6 (1.2)	10 (2.0)	329	14 (4.3)	2 (0.6)	4 (1.2)	6 (1.8)	148

We aim to continue our efforts in collecting comprehensive survey data through more active collaboration with the Japan Esophageal Society and other related institutions.
